# Development of a Strain-Specific Detection and Quantification Method for *Bifidobacterium animalis* subsp. *lactis* HN019 Using WGS-SNP Analysis and qPCR

**DOI:** 10.3390/microorganisms13071596

**Published:** 2025-07-07

**Authors:** Da Mao, Lei Zhao, Bo Zhao, Hongbin Xu, Qinghe Zhang

**Affiliations:** 1 Division of Chemical Metrology and Analytical Science, National Institute of Metrology, Beijing 100029, China; maoda@nim.ac.cn (D.M.); zhaobo@nim.ac.cn (B.Z.); zhangqh@nim.ac.cn (Q.Z.); 2 Key Laboratory of Milk and Dairy Products Detection and Monitoring Technology, State Administration for Market Regulation, Shanghai Institute of Quality Inspection and Technical Research, Shanghai 200233, China; xuhb@sqi.org.cn

**Keywords:** *Bifidobacterium animalis* subsp. *lactis*, strain-specific, identification, quantification, WGS-SNP

## Abstract

Accurate quantification of *Bifidobacterium animalis* subsp. *lactis* HN019, a clinically validated probiotic strain conferring immune modulation, gastrointestinal health, and gut barrier integrity benefits, is essential for diverse applications. To address the critical need for strain-specific detection, we developed a quantitative PCR (qPCR) assay targeting a unique single-nucleotide polymorphism (SNP) within the *gal*K gene, identified through comparative whole-genome sequencing (WGS) analysis of 31 *B. animalis* subsp. *lactis* strains. The assay exhibited exceptional specificity, distinguishing HN019 from 19 other *Bifidobacterium* strains. Sensitivity tests indicated a detection limit of 0.5 pg of DNA and 10^3^ CFU/mL of bacterial cells, making it suitable for industrial-scale applications. Additionally, the method exhibited strong repeatability, reproducibility across different qPCR platforms, and resistance to interference from high cell density of *B. animalis* subsp. *lactis* DSMZ 10140. Successful quantification of HN019 in complex multi-strain probiotic powders confirmed its practical reliability. This work establishes a rapid, robust, and scalable tool for precise probiotic strain tracking, addressing critical quality control and regulatory compliance needs within the rapidly expanding probiotic industry.

## 1. Introduction

Probiotics are live microorganisms that confer health benefits when administered in adequate quantities and have become integral to modern therapeutic strategies, particularly in the context of precision medicine and microbiome-targeted interventions [1]. Recent insights into host–microbiome interactions, such as the “gut–brain axis” and “gut–liver axis”, have expanded their therapeutic potential beyond gastrointestinal health to encompass neurological, metabolic, and immune-related disorders [2,3,4]. These advancements underscore the need for precise strain-level characterization to ensure consistency in probiotic efficacy and safety.

Among probiotics, *Bifidobacterium animalis* subsp. *lactis* HN019 exemplifies translational promise due to its exceptional gastrointestinal persistence [5] and biological stability [6]. Extensive research has demonstrated its ability to enhance intestinal barrier function, exclude pathogens, and reduce the severity of pediatric infections [7,8]. Systemically, HN019 modulates immune-metabolic pathways by balancing the COX-2/COX-1 ratio [9], suppressing inflammatory cascades [10], and ameliorating conditions like radiation-induced colitis [11]. Clinically, it shortens the duration of respiratory infection-induced fever and improves bowel motility [12,13]. Notably, HN019 has been shown to reduce the risk of viral infections in infants [14] and enhance cellular immune function in elderly populations [15], highlighting its broad applicability across age groups and supporting its use in infant formulas, functional foods, and therapeutics [16].

The efficacy and safety of probiotics are inherently strain-specific, necessitating accurate identification and quantification for product development, quality control, and regulatory compliance. However, existing methods for strain identification and quantification are limited by significant challenges. Colony morphology, while historically used, requires 3–5 days for analysis and lacks resolution for species differentiation, with phenotypes often influenced by environmental factors such as nutrient availability and incubation conditions [17]. Genetic markers like 16S rRNA sequencing enabled species-level identification but failed to resolve subspecies distinctions, such as differentiating *B. animalis* subsp. *lactis* from *B. animalis* [18]. The *atp*D gene (ATP synthase subunit beta) improved subspecies resolution, but it could not distinguish between strains due to high sequence conservation [19]. Molecular fingerprinting techniques like pulsed-field gel electrophoresis (PFGE) and random amplified polymorphic DNA (RAPD) have been employed for strain typing [20]; these techniques are often complex, equipment-intensive, and unsuitable for large-scale industrial applications. To address these challenges, whole-genome sequencing (WGS) has emerged as a transformative tool. WGS-based single-nucleotide polymorphism (SNP) analysis helps identify strain-specific genetic markers with unprecedented precision [21]. When combined with fluorescence quantitative PCR (qPCR), WGS-SNP analysis offers a practical and scalable approach for quality control and regulatory applications in probiotic products.

In this study, we focus on *B. animalis* subsp. *lactis* HN019, employing WGS-SNP analysis to identify strain-specific genetic markers. These markers are integrated with fluorescence quantitative PCR to develop a highly efficient, specific, and stable method for the quantification of HN019. Through the validation of the method’s specificity, sensitivity, repeatability, reproducibility, and resistance to interference, we establish a framework for strain-level detection that can be applied both in research settings and across industrial applications. This study provides technical support for the market regulation of probiotic foods and the healthy development of the industry.

## 2. Materials and Methods

### 2.1. SNP Analysis

SNPs were analyzed in 31 *B. animalis* subsp. *lactis* strains, including BB-12, Bi07, and others (Table 1), using whole-genome sequences retrieved from the NCBI database. The *B. animalis* subsp. *lactis* HN019 genome served as the reference. Sequence alignments were performed with MUMmer (version 3.23, https://mummer.sourceforge.net/, accessed on 8 February 2025) [22]. Initially, the nucmer utility [23] included with the MUMmer (version 3.23, https://mummer.sourceforge.net/, accessed on 8 February 2025) was used for global pairwise alignment of the genomes from the 30 strains against the HN019 genome, generating a comprehensive map of genomic similarities and differences. To improve alignment accuracy, the delta-filter program (MUMmer version 3.23, https://mummer.sourceforge.net/, accessed on 10 February 2025) was applied to remove low-confidence alignments and redundant genomic regions, retaining only high-quality data [24]. SNPs were identified using the show-snps tool in MUMmer (version 3.23, https://mummer.sourceforge.net/, accessed on 12 February 2025), extracting variant positions between the HN019 strain and the other 30 strains. The analyses were conducted by Majorbio (Shanghai, China).

### 2.2. Primer and Probe Design

A total of 10,357 SNPs were identified in the *B. animalis* subsp. *lactis* HN019 genome (Table S1). Among these, six variant sites were shared with the other 30 strains, and five of these differential SNPs, located within four single-copy genes, were selected for primer and probe design. Sequences were designed using Primer Express 3.0 (Applied Biosystems, Foster City, CA, USA) [25]. Comparative evaluation revealed that the TaqMan assay targeting the *gal*K SNP exhibited superior specificity for HN019 detection versus assays for other loci, showing no cross-reactivity with phylogenetically related subspecies strains. The sequences of the primers and probe used in this study are listed in Table 2 and were synthesized by Sangon Biotech (Shanghai, China).

### 2.3. DNA Extraction

Genomic DNA was extracted from the bacterial strains and samples using the Bacterial Genomic DNA Extraction Kit (No. DP302; Tiangen, Beijing, China), following the manufacturer’s instructions [26]. The extracted DNA was assessed by electrophoresis on a 1.0% agarose gel, and its concentration, 260/280 ratio, and 260/230 ratio were determined using a DS-11 spectrophotometer (DeNovix Inc., Wilmington, DE, USA) [27]. The DNA samples were then stored at −20 °C for further use.

### 2.4. Specificity

Primer and probe specificity were assessed using 20 different *Bifidobacterium* strains (Table 3). The qPCR reaction mixture consisted of 10 µL of Takara Prober Taq mix, 1 µL of upstream primer (5 µM), 1 µL of downstream primer (5 µM), 1 µL of probe (10 µM), 1 µL of DNA template, and double-distilled water (Beyotime Biotechnology, Beijing, China) to a final volume of 20 µL. The qPCR amplification was performed on the Roche LightCycler^®^ 480 platform (Roche Diagnostics GmbH, Mannheim, Germany) [28]. The thermal cycling conditions were as follows: Stage 1, 95 °C for 2 min; Stage 2, 95 °C for 5 s, 64 °C for 35 s, for a total of 40 cycles. Sterile double-distilled water (Beyotime Biotechnology, Beijing, China) was used as the blank control.

### 2.5. Stability During Passage

*B. animalis* subsp. *lactis* HN019 was passaged from 1 to 35 generations. DNA was extracted from bacterial suspensions at the 5th, 15th, 25th, and 35th generations, each at a cell density of 10^8^ CFU/mL. The DNA template was adjusted to a concentration of 50 ng/µL for qPCR amplification. Sterile double-distilled water (Beyotime Biotechnology, Beijing, China) was used as the blank control. The qPCR amplification was performed on the Roche LightCycler^®^ 480 platform (Roche Diagnostics GmbH, Mannheim, Germany). The differences in Cq values were analyzed to assess passage stability.

### 2.6. Sensitivity and Efficiency

Sensitivity was evaluated for both DNA concentration and cell density. For DNA concentration sensitivity, a 50 ng/µL DNA solution was serially diluted in 10-fold steps, followed by qPCR amplification. For bacterial cell density sensitivity, DNA was extracted from bacterial suspensions with concentrations ranging from 10^8^ to 10^3^ CFU/mL, followed by qPCR amplification. The bacterial cell densities of *B. animalis* subsp. *lactis* HN019 were measured using the Chinese National Standard GB 4789.34-2016 [29]. The qPCR amplification was performed on the Roche LightCycler^®^ 480 platform. Standard curves were constructed by plotting the logarithm of DNA concentration (Log [ng/µL]) or cell density (Log [CFU/mL]) against the Cq value using GraphPad Prism 9 [30]. The slope and R^2^ values were calculated, and amplification efficiency was determined using the qPCR Efficiency Calculator (Thermo Fisher Scientific, Waltham, MA, USA, https://www.thermofisher.cn/) [31].

### 2.7. Repeatability and Reproducibility

Repeatability was evaluated by conducting the experiment three times to test the consistency of the method. Reproducibility was assessed by performing experiments on two different qPCR platforms, Roche LightCycler^®^ 480 and ABI 7500 (Applied Biosystems, Foster City, CA, USA) [32]. The tests were conducted at three different DNA concentrations (50 ng/µL, 5 ng/µL, and 0.5 ng/µL), with each experiment performed in triplicate, using double-distilled water as the blank control.

### 2.8. Interference Resistance

The anti-interference experiment was designed to validate the specificity and sensitivity of the qPCR assay for quantifying *B. animalis* subsp. *lactis* HN019 in complex microbial matrices containing genetically similar strains. This validation is critical for ensuring accurate quantification in real-world scenarios, such as probiotic products or gut microbiota samples, where closely related subspecies may coexist at high concentrations. Bacterial cell densities of *B. animalis* subsp. *lactis* HN019 at concentrations ranging from 10^8^ to 10^3^ CFU/mL were mixed with an equal volume of a 10^8^ CFU/mL of *B. animalis* subsp. *lactis* DSMZ 10140. DNA was extracted from these mixtures, followed by qPCR amplification. The qPCR amplification was performed on the Roche LightCycler^®^ 480 platform. Each experiment was conducted in triplicate, with double-distilled water as the blank control.

### 2.9. Actual Sample Test

The developed quantification method for the HN019 strain was applied to analyze actual bacterial powder samples. The powder, containing *B. animalis* subsp. *lactis* HN019, *Lactobacillus acidophilus* NCFM, *Lacticaseibacillus rhamnosus* GG, and oligosaccharides, was prepared according to the Chinese National Standard GB 4789.34-2016 [29]. A 25 g sample was dissolved in 225 mL sterile physiological saline, followed by a 10-fold serial dilution, and the dilution factor was recorded. DNA was extracted from bacterial suspensions (10^3^ to 10^8^ CFU/mL) following the method described in Section 2.3. The qPCR amplification was performed on the Roche LightCycler^®^ 480 platform, with sterile physiological saline as the blank control. Standard curves were constructed by plotting the logarithm of cell density (Log [CFU/mL]) against the Cq value. In accordance with the Professional Standard of the People’s Republic of China for Entry-Exit Inspection and Quarantine SN/T 5642.3-2023 [33], the single-copy gene was used to establish equivalence between genomic copies and CFU, enabling direct reporting of results as CFU/g. The bacterial content of the product was calculated using the following formula:

M = 10^x^ × (A + B)/A × C

where

M = *B. animalis* subsp. *lactis* HN019 content in the bacterial powder (CFU/g);

A = mass of the sample taken for analysis (g);

B = volume of dilution solution (mL);

C = dilution factor of the sample suspension.

### 2.10. Statistical Analysis

Each experiment was performed thrice. The results were subjected to one-way ANOVA analysis and Tukey’s multiple comparisons test by Graphpad Prism (version 9.0), with *p* < 0.05 considered statistically significant.

## 3. Results

### 3.1. SNP Analysis and Specificity Test

**Figure 1:**
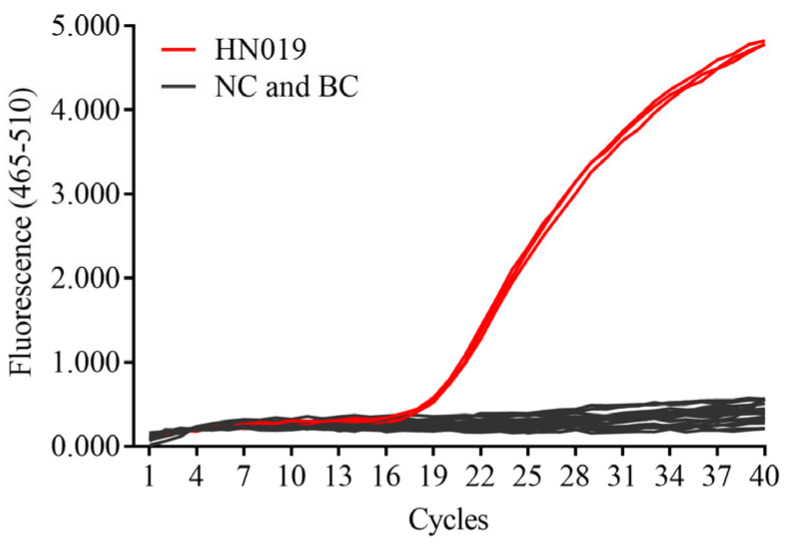
Primer specificity test for the *B. animalis* subsp. *lactis* HN019 strain. NC: Negative Control (strains other than HN019); BC: Blank Control (sterile double-distilled water).

Using the whole genome sequence of *B. animalis* subsp. *lactis* HN019 as a reference, we compared it with the whole-genome sequences of 30 other *B. animalis* subsp. *lactis* strains from the NCBI database. A total of 6 SNPs specific to the HN019 strain were identified. Among them, the SNP located in the *gal*K gene was selected for the design of primers and probes. After performing qPCR amplification, this SNP-based primer/probe pair was able to effectively differentiate *B. animalis* subsp. *lactis* HN019 from 19 other *Bifidobacterium* strains. As shown in Figure 1, the Cq values for the HN019 strain were 19.25, 19.27, and 19.07, while no significant amplification was observed for the other strains or the blank control. Based on the criteria for interpreting qPCR results outlined by Wang et al. [34], a Cq value below 35 was considered positive.

### 3.2. Passage Stability Test

As shown in Table 4, the Cq value standard deviation for the stability test of the HN019 strain, from the 5th to the 35th generation, was 0.24, with a relative standard deviation of 1.31%. In addition, the investigation revealed no statistically significant disparities in the outcomes of the evaluation between the 5th, 15th, 25th, and 35th generations. This indicates that the SNP site located in the *gal*K gene exhibits good stability throughout the passage process and can be used for the detection of *B. animalis* subsp. *lactis* HN019 in limited generations.

### 3.3. Sensitivity Test

**Figure 2:**
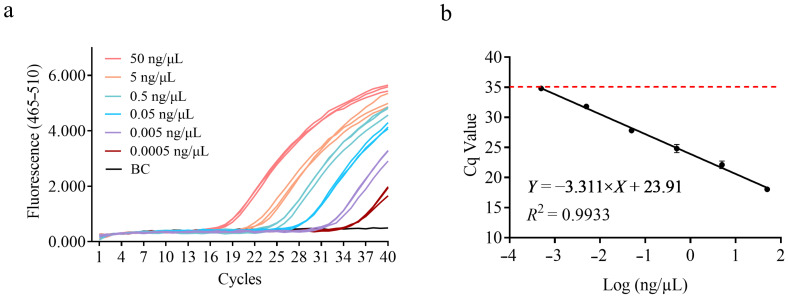
Sensitivity of the specific detection method for *B. animalis* subsp. *lactis* HN019 strain at varying DNA concentrations. (**a**) Amplification curve obtained using the Roche LightCycler^®^ 480 platform for the DNA concentration sensitivity test. (**b**) Standard curve constructed by plotting Cq values against DNA concentration.

To determine the sensitivity of this detection method, a DNA solution with a concentration of 50 ng/μL was serially diluted in 10-fold steps and subjected to qPCR amplification, as shown in Figure 2a. A standard curve was constructed with the logarithmic value of DNA concentration on the *x*-axis and the mean Cq value on the *y*-axis. From Figure 2b, the slope of the standard curve was −3.311, with an amplification efficiency of 100.46% and an *R*^2^ value of 0.9933 (Figure 2b). Based on the data in Figure 2, the detection limit for DNA concentration in this amplification system is approximately 0.5 pg, indicating that the detection method is highly sensitive.

**Figure 3:**
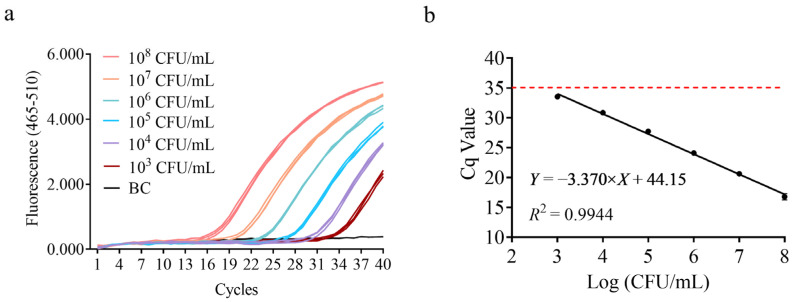
Cell density sensitivity test for specific detection method of *B. animalis* subsp. *lactis* HN019 strain. (**a**) Amplification curve obtained using the Roche LightCycler^®^ 480 platform for the bacterial cell density sensitivity test, BC: Blank Control (sterile double-distilled water). (**b**) Standard curve constructed by plotting Cq values against cell density.

To meet the needs of dairy companies for detecting lactic acid bacterial colony counts, quantitative results were correlated with bacterial colony-forming units (CFUs). A gradient dilution of *B. animalis* subsp. *lactis* HN019 bacterial suspension was prepared, DNA was extracted as the template, and qPCR amplification was performed. The amplification results showed that when the bacterial cell density was ≥10^3^ CFU/mL, the detection was positive, as shown in Figure 3. A standard curve was constructed with the logarithmic value of bacterial concentration on the x-axis and the average Cq value on the y-axis. The slope was −3.370, with an amplification efficiency of 98.03% and an *R*^2^ value of 0.9944. These results demonstrate that the sensitivity of this method for bacterial suspension concentration is 10^3^ CFU/mL, which meets the daily detection needs of dairy companies for 10^6^ CFU/mL [35].

### 3.4. Repeatability and Reproducibility Test

**Figure 4:**
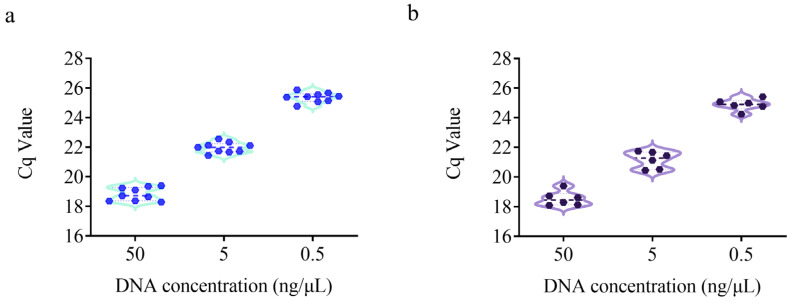
Repeatability (**a**) and reproducibility (**b**) test for specific detection method of *B. animalis* subsp. *lactis* HN019 strain. Violin plots were generated using GraphPad Prism 9, Median (dashed line), kernel density distribution (blue line or purple line) are shown. (**a**) Repeatability test, showing three trials with three replicates (dark blue dots) in each trial, for a total of 9 measurements. (**b**) Reproducibility test, showing experiments conducted on two different qPCR platforms (Roche LightCycler^®^ 480 and ABI 7500), for a total of 6 measurements (dark purple dots).

The repeatability and reproducibility of the detection method were evaluated using DNA samples at three different concentrations (50, 5, and 0.5 ng/μL). As shown in Figure 4a, the RSD for repeatability over time ranged from 1.30% to 2.38%. For reproducibility testing across two different qPCR platforms (Roche LightCycler^®^ 480 and ABI 7500), the RSD ranged from 1.55% to 2.65% (Figure 4b), which is well below the requirement that qPCR detection results should have an RSD value of less than 25% [36]. In the context of repeatability testing, no statistically significant variations were observed among the three test results at 50 ng/µL, 5 ng/µL, and 0.5 ng/µL. In the context of reproducibility testing, no statistically significant discrepancies were observed between the results obtained from two distinct platforms at concentrations of 50 ng/µL, 5 ng/µL, and 0.5 ng/µL. Therefore, the method demonstrated good repeatability and reproducibility and is suitable for use across different qPCR platforms.

### 3.5. Interference Resistance Test

**Figure 5:**
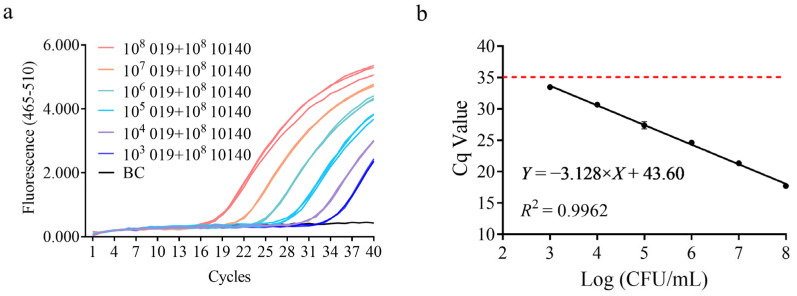
Interference resistance test for specific detection method of *B. animalis* subsp. *lactis* HN019 strain. (**a**) Amplification curve obtained using the Roche LightCycler^®^ 480 platform for the interference resistance test, BC: Blank Control (sterile double-distilled water). (**b**) Standard curve constructed by plotting Cq values against cell density.

To enhance the efficacy of probiotic products, a combination of target strains and other strains is often added to the products. Therefore, it is essential to verify the interference resistance of the HN019 strain detection method in the presence of closely related strains within the same subspecies. As shown in Figure 5, under the condition of adding 10^8^ CFU/mL of DSMZ 10140, the established detection method successfully quantified the HN019 strain, maintaining a sensitivity of 10^3^ CFU/mL. These results demonstrate that the method has strong resistance to interference, providing valuable data support for subsequent actual sample testing.

### 3.6. Quantification of HN019 Strain in Probiotic Powder Samples

**Figure 6:**
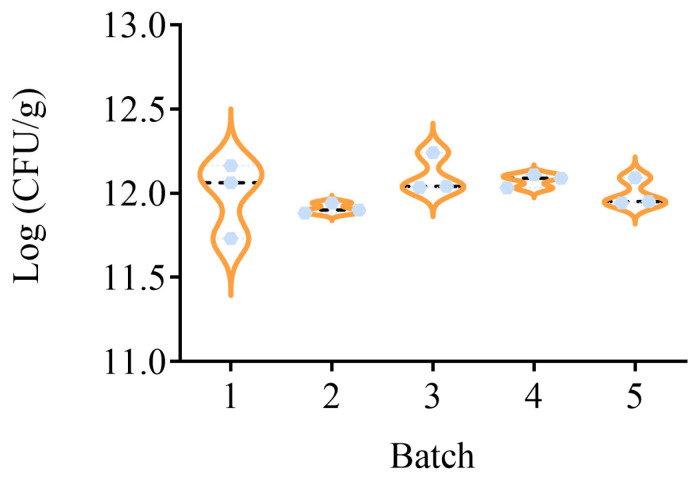
Validation of the specific detection method using five batches of multi-strain bacterial powder samples, including *B. animalis* subsp. *lactis* HN019, *L. acidophilus* NCFM, *L. rhamnosus* GG, and oligosaccharides. Violin plots were generated using GraphPad Prism 9, Median (dashed line), kernel density distribution (orange line) are shown. Each batch was tested three times, with the results of *B. animalis* subsp. *lactis* HN019 represented by light blue dots.

The strain-specific detection method was used to precisely quantify the HN019 strain in five batches of actual composite probiotic powder samples. Using the standard curve equation *Y* = −3.347 × *X* + 44.17, *R*^2^ = 0.9944, and the formula 2.9, the HN019 strain content in the five batches of samples was calculated to range from (11.91 ± 0.03) to (12.11 ± 0.12) Log (CFU/g), as shown in Figure 6. A subsequent investigation revealed that there was no significant difference in the content of HN019 in the five batches of probiotic powder samples. The results indicate that the method is equally applicable to composite probiotic powder samples containing large amounts of oligosaccharides and excipients.

## 4. Discussion

The selection of *B. animalis* subsp. *lactis* HN019 as the focal strain for this study stems from its well-established yet uniquely complex clinical profile and its prominence in commercially significant probiotic formulations. Originally isolated from dairy sources [37], HN019 has transcended its industrial origins to become a benchmark probiotic strain with documented, dose-dependent health benefits in humans. Critically, robust clinical trials have demonstrated their efficacy in enhancing innate immune responses, such as natural killer cell activity, and reducing the incidence of antibiotic-associated diarrhea. These specific immunomodulatory properties, coupled with their documented stability and persistence in the gut, have positioned HN019 as a cornerstone strain in precision probiotic interventions, particularly for vulnerable populations including infants and the elderly experiencing immunosenescence [7]. However, the very characteristic that makes HN019 valuable also presents a major challenge: its clinical outcomes are demonstrably formulation-dependent and inconsistent across studies, such as null effects observed in constipation trials [38]. This inconsistency underscores a fundamental limitation in the field: the inability to reliably confirm and quantify the presence and dose of the specific, clinically validated HN019 strain within complex probiotic products. Consequently, rigorous strain-specific quantification is not merely beneficial but essential for elucidating HN019’s true characteristics in vivo, ensuring product fidelity, validating clinical efficacy claims, and ultimately advancing evidence-based probiotic development.

While traditional culture-based methods provide total viable counts, they fundamentally lack the specificity to distinguish HN019 from closely related strains within the *B. animalis* subsp. lactis subspecies, especially in multi-strain products or those containing low target populations or complex matrices. This gap highlights a persistent problem: the lack of a truly strain-specific, sensitive, and robust quantification tool for HN019. Molecular approaches targeting genes like *tuf*, *Bal-23S*, or *Tal* have been successfully applied to quantify the related BB-12 strain [39,40,41] but critically fail to differentiate between HN019 and BB-12 due to insufficient genetic resolution. This gap highlights a persistent problem: the lack of a truly strain-specific, sensitive, and robust quantification tool for HN019.

With the rapid advancement of high-throughput sequencing technologies, the complete genome sequences of more *B. animalis* subsp. *lactis* strains have been completed, and genomic data are available through public databases, providing a solid foundation for the application of WGS-SNP analysis. By designing primers and probes for SNPs in the *gal*K gene, we successfully achieved specific detection of the HN019 strain. The selected SNP in *gal*K exhibited robust genetic stability across 35 serial passages, a critical validation for industrial scalability that ensures consistent detectability throughout product lifecycles. According to the criterion of Cq values below 35 for positive results in qPCR, the DNA sensitivity and cell density sensitivity of our quantitative detection method are 0.5 pg and 10^3^ CFU/mL, respectively. Additionally, the amplification efficiency exceeds 98%, falling within the optimal range (80–120%) [36], with an *R*^2^ value greater than 0.99, indicating excellent linearity. Evaluation of the method’s repeatability and reproducibility showed good consistency across different time intervals and platforms, with RSD values below 3%, confirming its high reliability and adaptability. To further assess the method’s resistance to interference, we conducted interference experiments by adding high concentrations of homologous strains. The results indicated that even with interference from other homologous strains and excipients, the method accurately quantifies the HN019 strain at 10^3^ CFU/mL, while traditional plate counting methods fail to achieve such specific quantification in multi-strain samples.

The accuracy and reliability of quality control for the HN019 strain, a key probiotic, directly affect consumer health. The quantitative analysis method proposed in this study, based on WGS-SNP analysis and qPCR, offers high specificity, strong repeatability, genetic stability, resistance to interference, and good equipment adaptability, providing regulators and manufacturers with a gold-standard tool to verify HN019 strain identity, viability, and dose claims in commercial products, safeguarding against mislabeling and subpotency. Moreover, the SNP-based design paradigm establishes a blueprint for developing strain-specific assays for other high-value probiotics, advancing the field toward truly personalized microbial therapeutics.

## 5. Conclusions

As the probiotic market rapidly expands, consumers’ demands for the quality and safety of probiotic products are increasing. Accurate and reliable strain detection methods have become one of the key technologies driving the sustainable development of the probiotic industry. This study proposes a high-precision HN019 strain quantification method based on WGS-SNP analysis and quantitative PCR technology, providing scientific and reliable technical support for quality control of probiotic products. Our WGS-SNP-qPCR platform not only unlocks rigorous research into HN019’s bioactivity but also sets a new standard for quality control in the rapidly evolving probiotic industry, ensuring that evidence-based health claims translate into reliable consumer benefits.

## Figures and Tables

**Table 1:** Strains used for WGS-SNP analysis.

Species	Strain	Assembly Number	Scaffold Number	Genome Size (Mb)
*B. animalis* subsp. *lactis*	HN019 *	GCA_003606305.1	1	1.935423
	19-D-1	GCA_020309945.1	1	1.963057
	AD011	GCA_000021425.1	1	1.933695
	ATCC 27673	GCA_000471945.1	1	1.963012
	B420	GCA_000277325.1	1	1.938595
	BAMA-B06	GCA_028463865.1	1	1.944252
	BB-12	GCA_000025245.2	1	1.944152
	BF052	GCA_000818055.1	1	1.938624
	BGI-N3	GCA_040084745.1	1	1.944238
	BI040	GCA_038086955.1	1	1.944141
	Bi-07	GCA_000277345.1	1	1.938822
	Bl-04	GCA_000022705.1	1	1.938709
	Bl12	GCA_000414215.2	1	1.944036
	BLC1	GCA_000224965.2	1	1.938583
	CGMCC1.15623	GCA_044866785.1	1	1.944145
	CNCM I-2494	GCA_000220885.1	1	1.943113
	DSM 10140	GCA_000022965.1	1	1.938483
	DSM 15954	GCA_021018785.1	1	1.944152
	GOLDGUT-BB18	GCA_034561975.1	1	1.944144
	H1	GCA_016835115.1	1	1.944352
	H3	GCA_016835135.1	1	1.944351
	HOM2120	GCA_044502405.1	1	1.946900
	i797	GCA_019576095.1	1	1.943538
	IDCC4301	GCA_003428375.1	1	1.944141
	KLDS 2.0603	GCA_000816205.1	1	1.946899
	LPL-RH	GCA_030284625.1	1	1.944223
	MH-02	GCA_029167605.1	1	1.944290
	S7	GCA_003390755.1	1	1.944072
	SF	GCA_029542645.1	1	1.944374
	TCI604	GCA_036923305.1	1	1.944134
	V9	GCA_000092765.1	1	1.944050

*: reference genome.

**Table 2:** The sequences of primers and probe.

Gene	The Sequences of Primers and Probe	Product Length (bp)
*gal*K	TGATATGGCGAATGCTTGCA	61
	CGGCTTGTGTGTCGTCATG	
	FAM-ACCCTTTTCATTTCCCTGC-MGB	

**Table 3:** Strains used for specificity test.

No.	Species	Strain
1	*B. animalis* subsp. *lactis*	HN019
2		Bi-07
3		Bl-04
4		BB12
5		DSMZ 10140
6		CICC 21718
7		ATCC 25527
8		ATCC 27673
9	*B. animalis*	ATCC 27672
10		CICC 6165
11		CICC 6250
12	*B. adolescentis*	ATCC 15703
13	*B. longum* subsp. *infantis*	ATCC 15697
14	*B. breve*	ATCC 15700
15		CICC 6185
16		CICC 6181
17	*B. bifidum*	CICC 6173
18	*B. longum*	CICC 6207
19		CICC 6199
20		Bl05

**Table 4:** Stability of specific amplification during serial passages.

Number of Generations	Cq Value			Mean Cq Value	SD	RSD (%)
5	18.88	18.78	18.98	18.88	0.24	1.31
15	18.92	18.86	18.27	18.68		
25	18.73	18.38	18.89	18.67		
35	17.98	18.4	18.5	18.29		

## Data Availability

The full genome data of 31 *B. animalis* subsp. *lactis* can be found in NCBI GenBank under the assembly number listed in Table 1.

## Supplementary Materials

The following supporting information can be downloaded at: https://www.mdpi.com/article/10.3390/microorganisms13071596/s1. Table S1: Strains used for genomic comparison and specific loci information.
